# Supplementary material for: Nursing students’ attitude on the practice of e-learning: A cross-sectional survey amid COVID-19 in Nepal
Source: PLoS One. 2021 Jun 24;16(6):e0253651. doi: 10.1371/journal.pone.0253651 (PMC8224981; doi:10.1371/journal.pone.0253651)
Supplement: S1 Tool — (DOCX) [file pone.0253651.s001.docx]

**QUESTIONNAIRE**

**Research Title**: ***Nursing students’ attitude on the practice of e-Learning in selected medical colleges: A Cross sectional survey amid Covid 19 in Nepal***

The questionnaire contains four parts:

PART 1: Questionnaire related to socio demographic information

PART 2: Questionnaire related to advantages and disadvantages of e-learning

PART 3: Likert scale related to level of acceptance of e-learning

PART 4: Standard Likert scale measuring the attitude of students regarding e-learning.

**Part 1: Socio Demographic Information**

1. Age:
2. Residence: a. Urban b. Rural
3. Monthly family income:
4. Name of College:
5. Year of study:
6. Gadgets/device used in learning:
7. Mobile
8. Computer
9. Laptop
10. Tablet
11. Source of internet:
12. Previously participated in any online courses: a. Yes b. No

**Part 2: Questionnaire related to Advantages and Disadvantages of E-Learning**

1. What are the advantage of using e-learning? (***Multiple response)***
2. Learning in your own pace
3. Ability to stay at home
4. Classes’ interactivity
5. Ability to record a meeting
6. Comfortable
7. Remote access
8. Reduce cost of accommodation and transportation
9. Others:
10. What are the disadvantages of using e-learning?
11. Reduced interaction with patients
12. Poor learning condition at home
13. Lack of self-discipline
14. Social isolation
15. Internet problems
16. Technical issues
17. Poor interaction with facilitators
18. Other:

**Part III: Likert scale related to level of acceptance of e-learning in relation to traditional face to face learning method**

| **S.N** | **Statements** | **SE** | **E** | **N** | **I** | **SI** |
| --- | --- | --- | --- | --- | --- | --- |
| 1 | E learning is better than traditional learning |  |  |  |  |  |
| 2* | E learning is not secured. |  |  |  |  |  |
| 3 | E-learning is better than traditional learning in terms of increasing knowledge. |  |  |  |  |  |
| 4 | E-learning is better than traditional learning in terms of increasing skills |  |  |  |  |  |
| 5 | E-learning is better than other learning in terms of achieving social competencies |  |  |  |  |  |
| 6 | E-learning is more enjoyable than face to face learning |  |  |  |  |  |
| 7* | E learning makes the participants less active than in the face to face learning. |  |  |  |  |  |
| 8 | E-learning is an innovative idea and must be encouraged. |  |  |  |  |  |

*Where, SE= Strongly effective, E=Effective, N=Neutral, I=Ineffective, SI=Strongly ineffective*

**Negative statement*

**Part V: Likert scale measuring attitude of the nursing students regarding e-learning**

| **S.N** | **Statements** | | **SD** | **DA** | **N** | **A** | **SA** |
| --- | --- | --- | --- | --- | --- | --- | --- |
| 1. | **Perceived usefulness** | |  |  |  |  |  |
|  | a. | E-learning can solve many of the educational problems. |  |  |  |  |  |
|  | b. | E-learning saves time. |  |  |  |  |  |
|  | c. | E-learning improves access to learning material. |  |  |  |  |  |
|  | d. | E-learning helps me to achieve better results. |  |  |  |  |  |
|  | e. | E-learning increase learner’s engagement in learning. |  |  |  |  |  |
|  | f. | E- learning improve teacher and students interaction |  |  |  |  |  |
|  | g. | E-learning increase my understanding of concept |  |  |  |  |  |
|  | h.* | E-learning has created more problems than it solved |  |  |  |  |  |
|  | i.* | E-learning is too time consuming to use. |  |  |  |  |  |
|  | j.* | E-learning has had little impact on me |  |  |  |  |  |
|  | k. | E-learning is as informative as the teacher |  |  |  |  |  |
|  | l.* | E-learning will never replace other forms of teaching and learning. |  |  |  |  |  |
|  | m. | E-learning help to reinforce my knowledge. |  |  |  |  |  |
|  | n. | E-learning help me to organize my work |  |  |  |  |  |
|  | o. | E-learning help me to catch up missed lectures |  |  |  |  |  |
|  | p. | E-learning increase my effectiveness to create presentations. |  |  |  |  |  |
|  | q. | E-learning increase my research capability. |  |  |  |  |  |
|  | r. | Universities should adopt e-learning for their students. |  |  |  |  |  |
| 2. | **Intention to adopt e-learning** | |  |  |  |  |  |
|  | a.* | E-learning makes me uncomfortable because I don’t understand it |  |  |  |  |  |
|  | b.* | E-learning is a de-humanizing process of learning. |  |  |  |  |  |
|  | c.* | I dislike the idea of using E-learning. |  |  |  |  |  |
|  | d.* | I am not in favor of E-learning as it leads to social isolation. |  |  |  |  |  |
|  | e.* | E-learning doesn’t interest me. |  |  |  |  |  |
|  | f. | I plan to participate in future e-learning courses |  |  |  |  |  |
|  | g. | I plan to buy a computer to be able to follow lectures notes online |  |  |  |  |  |
|  | h. | Using E-learning makes learning fun. |  |  |  |  |  |
|  | i. | I don’t know what I would do without E-learning. |  |  |  |  |  |
| 3. | **Ease of learning** | |  |  |  |  |  |
|  | a.* | Using E-learning is more difficult than using the library |  |  |  |  |  |
|  | b.* | I can’t read the lectures notes through the web |  |  |  |  |  |
|  | c.* | I can’t learn courses through the web. |  |  |  |  |  |
|  | d.* | It is difficult to acquire any significant information by using internet. |  |  |  |  |  |
|  | e.* | It is difficult to express my thoughts by writing through E- learning. |  |  |  |  |  |
|  | f.* | I find that using the internet make me slow |  |  |  |  |  |
|  | g.* | I feel we are becoming slaves to technology. |  |  |  |  |  |
|  | h.* | My interaction with E-learning is not understandable |  |  |  |  |  |
| 4. | **Technical support** | |  |  |  |  |  |
|  | a. | My institute has an updated website. |  |  |  |  |  |
|  | b. | My institute facilitates e-learning training program |  |  |  |  |  |
|  | c. | My institute has adequate technology for e-learning. |  |  |  |  |  |
|  | d. | I seek technical assistance from college support services. |  |  |  |  |  |
| 5. | **Learning stressor** | |  |  |  |  |  |
|  | a.* | Feel anxious about my ability to use e learning effectively. |  |  |  |  |  |
|  | b.* | Slow internet connections stress me |  |  |  |  |  |
|  | c.* | I feel pressured by my teachers to use E-learning for my research/ learning activities |  |  |  |  |  |
| 6. | **E-distant use of e- learning** | |  |  |  |  |  |
|  | a. | E-learning should be offered fully online to reach students living in remote areas. |  |  |  |  |  |
|  | b. | E-learning should be used to reduce travel related stress. |  |  |  |  |  |
|  | c. | E-learning should be adopted to allow married students to balance family and Study demands |  |  |  |  |  |
|  | d. | E-learning should be adopted to allow working students to study from home. |  |  |  |  |  |

*Where, SA= Strongly agree, A=Agree, N=Neutral, D=Disagree, SD=Strongly disagree*

**Negative statements*
