# Supplementary material for: Nursing students’ attitude on the practice of e-learning: A cross-sectional survey amid COVID-19 in Nepal
Source: PLoS One. 2021 Jun 24;16(6):e0253651. doi: 10.1371/journal.pone.0253651 (PMC8224981; doi:10.1371/journal.pone.0253651)
Supplement: Attachment — Submitted filename: PONE-D-21-00551_reviewer.pdf [file pone.0253651.s004.pdf]

PLOS ONE

Nursing students’ attitude on the practice of e-learning: A Cross-sectional survey amid Covid 19

--Manuscript Draft--

**Manuscript Number:**	PONE-D-21-00551
**Article Type:**	Research Article
**Full Title:**	Nursing students’ attitude on the practice of e-learning: A Cross-sectional survey amid Covid 19
**Short Title:**	Nursing students' attitude on the practice of e-learning amid Covid-19
**Corresponding Author:**	Pratima Thapa<br>College of Medical Sciences<br>Bharatpur, Chitwan NEPAL
**Keywords:**	Attitude, nursing sudents, e-learning, Covid-19, Nepal
**Abstract:**	Background<br>In present days, the use of information technology in the field of education is unquestionable. The mounting advancement of IT has changed the scenario of education and with the emergence of the current Covid-19 situation, it has surely provided a solution to most of our educational needs when all educational institutions remained shut down due to the pandemic.<br>This study aims to identify the attitude of the nursing students’ towards the practice of e-learning amidst covid-19.<br>Methods: A descriptive web-based cross-sectional study was conducted among nursing students with a sample size of 470. A self-administered validated questionnaire along with a standard tool to measure the attitude was used for data collection. Data were analyzed using SPSS.<br>Results: The mean ± SD age of the respondents was 20.91± 1.55. The majority (76.4%) of the respondents used mobile for their study and 90.4% used Wi-Fi as for the internet source. The main advantage of e-learning was stated as the ability to stay at home (72.1%) followed by the reduced cost of accommodation and transport (51.3%) whereas the internet problem (81.7%) was the major disadvantage followed by technical issues (65.5%). Only about 34% of the students found E-learning as effective to traditional face to face learning. The mean scores for perceived usefulness, intention to adopt, and distant use of e-learning were 3.06, 3.07, and 3.82 which is much more in comparison to ease of learning, technical support, and learning stressors where the scores are 2.91, 2.90, and 2.53 respectively. Overall, 58.9% had a favorable attitude regarding e-learning. There was no significant association of attitude regarding e-learning with age, residence, college, year of study, and being ever participated in e-learning.<br>Conclusion: Almost half of the nursing students had a positive attitude regarding e-learning. The majority of the students had internet problems, technological issues, and inadequate interaction with the patients. If e-learning can be made user-friendly with programs that can enhance practical learning abilities then e-learning can be the vital alternative method of teaching and learning in the nursing field.
**Order of Authors:**	Pratima Thapa
	Suman Lata Bhadari
	Sharada Pathak
**Additional Information:**	
**Question**	**Response**
**Financial Disclosure**<br><br>Enter a financial disclosure statement that describes the sources of funding for the work included in this submission. Review	The author received no specific funding for this work.

the submission guidelines for detailed requirements. View published research articles from *PLOS ONE* for specific examples.<br><br>This statement is required for submission and **will appear in the published article** if the submission is accepted. Please make sure it is accurate.<br><br>**Unfunded studies**<br>Enter: *The author(s) received no specific funding for this work.*<br><br>**Funded studies**<br>Enter a statement with the following details:<br>• Initials of the authors who received each award<br>• Grant numbers awarded to each author<br>• The full name of each funder<br>• URL of each funder website<br>• Did the sponsors or funders play any role in the study design, data collection and analysis, decision to publish, or preparation of the manuscript?<br>• **NO** - Include this sentence at the end of your statement: *The funders had no role in study design, data collection and analysis, decision to publish, or preparation of the manuscript.*<br>• **YES** - Specify the role(s) played.<br><br>* typeset	
**Competing Interests**<br><br>Use the instructions below to enter a competing interest statement for this submission. On behalf of all authors, disclose any competing interests that could be perceived to bias this work—acknowledging all financial support and any other relevant financial or non-financial competing interests.<br><br>This statement **will appear in the published article** if the submission is accepted. Please make sure it is accurate. View published research articles from *PLOS ONE* for specific examples.	The author have declared that no competing interests exist.

Powered by Editorial Manager® and ProduXion Manager® from Aries Systems Corporation

**NO authors have competing interests**<br><br>Enter: *The authors have declared that no competing interests exist.*<br><br>**Authors with competing interests**<br><br>Enter competing interest details beginning with this statement:<br><br>*I have read the journal's policy and the authors of this manuscript have the following competing interests: [insert competing interests here]*<br><br>* typeset	
**Ethics Statement**<br><br>Enter an ethics statement for this submission. This statement is required if the study involved:<br><br>• Human participants<br>• Human specimens or tissue<br>• Vertebrate animals or cephalopods<br>• Vertebrate embryos or tissues<br>• Field research<br><br>Write "N/A" if the submission does not require an ethics statement.<br><br>General guidance is provided below. Consult the submission guidelines for detailed instructions. **Make sure that all information entered here is included in the Methods section of the manuscript.**	Ethical consideration: Formal permission was obtained from the Institutional Review Committee of College of Medical Sciences- Teaching Hospital (COMSTH-IRC), Bharatpur-10 (Ref no. 2020-079, NHRC ref no. 2586). Written permission from the administrative section of all 4 colleges was obtained for data collection. Informed consent was obtained from all respondents to ensure their rights. The purpose and objectives of the study were clearly explained to all the respondents. Privacy and confidentiality of records was strictly maintained throughout the study

Powered by Editorial Manager® and ProduXion Manager® from Aries Systems Corporation

**Format for specific study types**<br><br>**Human Subject Research (involving human participants and/or tissue)**<br>• Give the name of the institutional review board or ethics committee that approved the study<br>• Include the approval number and/or a statement indicating approval of this research<br>• Indicate the form of consent obtained (written/oral) or the reason that consent was not obtained (e.g. the data were analyzed anonymously)<br><br>**Animal Research (involving vertebrate animals, embryos or tissues)**<br>• Provide the name of the Institutional Animal Care and Use Committee (IACUC) or other relevant ethics board that reviewed the study protocol, and indicate whether they approved this research or granted a formal waiver of ethical approval<br>• Include an approval number if one was obtained<br>• If the study involved *non-human primates*, add *additional details* about animal welfare and steps taken to ameliorate suffering<br>• If anesthesia, euthanasia, or any kind of animal sacrifice is part of the study, include briefly which substances and/or methods were applied<br><br>**Field Research**<br><br>Include the following details if this study involves the collection of plant, animal, or other materials from a natural setting:<br>• Field permit number<br>• Name of the institution or relevant body that granted permission	
**Data Availability**<br><br>Authors are required to make all data underlying the findings described fully available, without restriction, and from the time of publication. PLOS allows rare exceptions to address legal and ethical concerns. See the PLOS Data Policy and FAQ for detailed information.	Yes - all data are fully available without restriction

A Data Availability Statement describing where the data can be found is required at submission. Your answers to this question constitute the Data Availability Statement and **will be published in the article**, if accepted.<br><br>**Important:** Stating 'data available on request from the author' is not sufficient. If your data are only available upon request, select 'No' for the first question and explain your exceptional situation in the text box.<br><br>Do the authors confirm that all data underlying the findings described in their manuscript are fully available without restriction?	
**Describe where the data may be found in full sentences. If you are copying our sample text, replace any instances of XXX with the appropriate details.**<br><br>• If the data are **held or will be held in a public repository**, include URLs, accession numbers or DOIs. If this information will only be available after acceptance, indicate this by ticking the box below. For example: *All XXX files are available from the XXX database (accession number(s) XXX, XXX.).*<br>• If the data are all contained **within the manuscript and/or Supporting Information files**, enter the following: *All relevant data are within the manuscript and its Supporting Information files.*<br>• If neither of these applies but you are able to provide **details of access elsewhere**, with or without limitations, please do so. For example:<br><br>*Data cannot be shared publicly because of [XXX]. Data are available from the XXX Institutional Data Access / Ethics Committee (contact via XXX) for researchers who meet the criteria for access to confidential data.*<br><br>*The data underlying the results presented in the study are available from (include the name of the third party*	The datasets used and analyzed during the current study are available from the corresponding author on reasonable request.

and contact information or URL).<br>• This text is appropriate if the data are owned by a third party and authors do not have permission to share the data.<br><br>* typeset	
Additional data availability information:	

Powered by Editorial Manager® and ProduXion Manager® from Aries Systems Corporation

Manuscript

Click here to access/download;Manuscript;manuscript-elearning - Copy.docx

Nursing students' attitude on the practice of e-learning: A Cross-sectional survey amid Covid 19.

Pratima Thapa[1], Suman Lata Bhandari[2], Sharada Pathak[3]

[1]Lecturer, Department of Nursing, Maternal Health Nursing, College of Medical Sciences, Kathmandu University, Chitwan, Nepal

[2] Lecturer, Department of Nursing, Medical-Surgical Nursing, Manmohan Memorial Institute of Health Sciences, Tribhuwan University, Kathmandu

[3] Senior Lecturer, Department of Nursing, Medical-Surgical Nursing, Chitwan Medical College, Tribhuwan University, Chitwan, Nepal

Correspondence: Pratima Thapa, Department of Nursing, Maternal Health Nursing, College of Medical Sciences, Kathmandu University, Chitwan, Nepal

Tel: +9771 9849884273

Email: pratiimathapa@gmail.com

ORCID ID: https://orcid.org/0000-0003-2709-4986

Abstract

Background

In present days, the use of information technology in the field of education is unquestionable. The mounting advancement of IT has changed the scenario of education and with the emergence of the current Covid-19 situation, it has surely provided a solution to most of our educational needs when all educational institutions remained shut down due to the pandemic. This study aims to identify the attitude of the nursing students' towards the practice of e-learning amidst covid-19.

Methods: A descriptive web-based cross-sectional study was conducted among nursing students with a sample size of 470. A self-administered validated questionnaire along with a standard tool to measure the attitude was used for data collection. Data were analyzed using SPSS.

Results: The mean ± SD age of the respondents was 20.91± 1.55. The majority (76.4%) of the respondents used mobile for their study and 90.4% used Wi-Fi as for the internet source. The main advantage of e-learning was stated as the ability to stay at home (72.1%) followed by the

reduced cost of accommodation and transport (51.3%) whereas the internet problem (81.7%) was the major disadvantage followed by technical issues (65.5%). Only about 34% of the students found E-learning as effective to traditional face to face learning. The mean scores for perceived usefulness, intention to adopt, and distant use of e-learning were 3.06, 3.07, and 3.82 which is much more in comparison to ease of learning, technical support, and learning stressors where the scores are 2.91, 2.90, and 2.53 respectively. Overall, 58.9% had a favorable attitude regarding e-learning. There was no significant association of attitude regarding e-learning with age, residence, college, year of study, and being ever participated in e-learning.

Conclusion: Almost half of the nursing students had a positive attitude regarding e-learning. The majority of the students had internet problems, technological issues, and inadequate interaction with the patients. If e-learning can be made user-friendly with programs that can enhance practical learning abilities then e-learning can be the vital alternative method of teaching and learning in the nursing field.

Keywords

Attitude, nursing students, e-learning, Covid 19, Nepal

Background

The pandemic spread of Novel Coronavirus, also known as COVID-19, significantly disrupted every aspect of human life, including education. The alarming spread of the virus caused havoc in the educational system forcing educational institutions to shut down. (1) More than 1.5 billion students and youth across the planet got affected by school and university closures due to the COVID-19 outbreak. (2) In Nepal also, this pandemic steered the shutting down of all educational institutions for an unknown period of time. (3) With the beginning of the lockdown to control the Covid-19 outbreak, many educational institutions started switching from traditional classroom teaching to online teaching to cope with the adverse situation. Tribhuvan University (TU), the largest and oldest university in Nepal, also officially endorsed the virtual class model along with a guideline and circulated a notice among its institutions. Similarly, the Ministry of Education, Science, and Technology appealed to stakeholders to start classes through alternative systems. (4)

There are several studies based on the significance and efficacy of the implementation of e-learning. There are numerous reasons for its overall acceptability; a few of them particularly applicable in the case of learners are its ease of use, flexibility, and better control over the

environment. However, regardless of its several advantages, there are quite a few limitations of e-learning such as social isolation, lack of student-teacher interaction and connectivity problems, etc.(5)

As the schools and colleges got shut down for an indefinite period, both educational institutions and students were experimenting with ways to complete their prescribed syllabi in the stipulated time frame in line with the academic calendar. These measures certainly caused a degree of inconvenience, but it also encouraged new examples of educational innovation using digital interventions. This is a silver lining on a dark cloud considering the sluggish pace of reforms in academic institutions, which continues with millennia-old lecture-based approaches in teaching, ingrained institutional biases, and obsolete classrooms. (6)

The nationwide lockdown compelled the majority of the institutions and their teachers to run their classes online, mostly by using Zoom software, google meet, etc. The evolving trend in the Nepali education system has thus made Nepali teachers, students, and parents familiar with the online classes. (8)

Considering online education has prevailed in the medical field too where learning used to be mostly done through the principle of hands-on knowledge, both teachers and students are still in the process of getting accustomed to the new system. At this very point, it is important to know the students' opinions and viewpoints regarding this virtual approach to teaching and learning. The review of various works of literature has identified and accessed the perception and attitudes of the students towards e-learning during the covid-19 pandemic situation. So, based on this researcher is interested in carrying out a study regarding the attitude of the nursing students towards e-learning. The study is even more relevant that in Nepal the system of e-learning was never been tried at this large level and this is like a massive social experiment and that too also in a nursing field where learning most of the time takes place in labs and wards for the understanding of the real scenario.

Methods

The descriptive web-based cross-sectional study design was used in the study. Four nursing colleges of different universities were selected. The selected colleges were College of Medical Sciences- Kathmandu University (CMS-KU), Chitwan Medical College-Tribhuwan University

(CMC-TU), Pokhara Nursing College-Pokhara University (PU), and Shree Medical and Technical College-Purbanchal University (SMTC-PurU). The total duration of the study was 3 months from August to October 2020. The study population for this study comprised all the B.Sc. nursing students of 1st, 2nd, 3rd, and 4th year. The total enumerative sampling method was used to determine the sample size. The total number of students was 482 from which response was obtained only from 470 students. Those who were willing to participate in the study were included in the study as inclusion criteria.

A Self-administered web-based questionnaire was developed through a literature search. The questionnaire consisted of 4 parts where parts 1, 2, and 3 were developed by the researchers themselves and part 4 was adapted from previously published studies with authors' permission. (9)

PART 1: Questionnaire related to socio-demographic information (age, college, year of study, gadgets used in e-learning, sources of the internet, and previous experience)

PART 2: Questionnaire related to advantages and disadvantages of e-learning (sets of options were given from which they could choose as many as were true to them and also they could write if they had any other felt advantages or disadvantages)

PART 3: Likert scale related to the effectiveness of e-learning to traditional face to face learning method (from where the students had to compare e-learning with traditional learning using Likert scale 1=strongly effective to 5=strongly ineffective)

PART 4: Standard Likert scale measuring the attitude of students regarding e-learning. (10)
The scale has 6 domains: Perceived usefulness, intention to adopt e-learning, ease of e-learning use, technical support, e-learning stressors, and pressure to use e-learning ranging from strongly disagree=1 to strongly agree=5. There are a total of 46 items with 26 positive items and 20 negative items. To assess the overall attitude, the mean score of the 5-point Likert scale was considered.
The questionnaire was prepared in google docs and the link was sent to the students through the mail, WhatsApp, and Viber groups.

Ethical consideration: Formal permission was obtained from the Institutional Review Committee of College of Medical Sciences- Teaching Hospital (COMSTH-IRC), Bharatpur-10 (Ref no. 2020-

079, NHRC ref no. 2586). Written permission from the administrative section of all 4 colleges was obtained for data collection. Informed consent was obtained from all respondents to ensure their rights. The purpose and objectives of the study were clearly explained to all the respondents. Privacy and confidentiality of records was strictly maintained throughout the study

The validity of the instrument was maintained by extensive literature review and consultation from subject experts. The reliability of the instrument was examined for internal consistency after pre-testing in 10% of a similar type of estimated population. The reliability score of the Likert scale measuring the attitude of the nursing students towards e-learning was 0.69, 0.68, 0.65, 0.56, 0.66, and 0.57 for the domains: perceived usefulness, intention to adopt, ease, technical support, e-learning stressors, and pressure to use e-learning respectively.

All collected data were checked, reviewed, coded, and organized for their accuracy, completeness, and consistency. Data were analyzed using the Statistical Package for Social Science (SPSS) version 16. Data were analyzed and interpreted in terms of descriptive statistics (frequency, median, mean, percentage, and standard deviation) and inferential statistics, chi-square was used to assess the relationship of the attitude of respondents regarding e-learning with the selected sociodemographic variables. Overall attitude was categorized as favorable and unfavorable from the mean score of the 5-point Likert scale.

Results

Sociodemographic data

Of the total respondents, the majority (57.9%) were from the age group 20 to 25 with a mean ± SD age of 20.91 ± 1.55 years. The majority (83.2%) were from an urban area. Most (68.9%) had their family income of 15-50 thousand. It was found that 76.45% of the respondents use mobile for their e-learning and 90.4% use Wi-Fi as a source for the internet. The majority 91.1% of the participants had never participated in e-learning before this pandemic.

Table 1: Sociodemographic characteristics of the respondents (n=470)

Variables	Frequency	Percentage
**Age of the respondents** ***(Mean ± SD age: 20.91 ± 1.55)***		
16 to 20	195	41.5

>20 to 25	272	57.9
>25 to 30	3	0.6
**Residence**		
Rural	79	16.8
Urban	391	83.2
**Monthly family income**		
15,000 to 50,000	324	68.9
>50,000 to 1 Lakhs	117	24.9
>1 Lakhs	29	6.2
**Name of College**		
Pokhara University, PU	145	30.9
College of Medical Sciences, KU	126	26.8
Chitwan Medical College, TU	100	21.3
Shree Medical College, Purbanchal University	99	21.1
**Year of Study**		
1st Year	101	21.5
2nd Year	124	26.4
3rd Year	134	28.5
4th Year	111	23.6
**Gadgets Used**		
Mobile	359	76.4
Computer	6	1.3
Laptop	103	21.9
Tablet	2	0.4
**Source of Internet**		
Wi-Fi	425	90.4
Telephone line	22	4.7
Mobile Data	23	4.9
**Ever participated in e-learning**		
Yes	42	8.9
No	428	91.1

Advantage and disadvantages of e-learning

The majority (72.1%) of the respondents responded the advantage of e-learning as the ability to stay at home followed by the reduced cost of accommodation and transport (51.3%), and the ability to record the meeting (38.1%). Regarding the disadvantages, the majority (81.7%) find it difficult due to internet problems followed by technical issues (65.5%) and reduced interaction with the patients (55.1%).

Table 2: Advantages and disadvantages of e-learning

**Advantages**	**Frequency**	**Percentage**
Ability to stay at home	339	72.1
Classes interactivity	68	14.5
Ability to record meeting	179	38.1
Comfortable environment	156	33.2
Remote access	42	8.9
Reduce the cost of accommodation and transport	241	51.3
Learning in your environment	1	0.2
Time-saving	1	0.2
Helps to screenshot many beneficial slides	1	0.2
**Disadvantages**		
Reduced interaction with patients	259	55.1
Poor learning condition at home	185	39.4
Lack of self-discipline	112	23.8
Reduced socialization	103	21.9
Internet problems	384	81.7
Technical issues	308	65.5
Poor interaction with facilitators	213	45.3
Eye problem	1	0.2

Fig- 1 shows the Effectiveness of e-learning to traditional face to face learning method where E-learning was found to be less effective than traditional face to face learning with a mean score of 2.62.

The attitude of respondents regarding e-learning

Perceived usefulness

With a mean score of 3.21, the majority (39.5%) of the respondents felt that e-learning can solve many of the educational problems. More than half (64.7%) said that it helps in saving their time and 40.8% expressed that e-learning improves their access to other learning material. However, achieving the better result increased learner's engagement in learning, improved teacher and student interaction, and improving understanding of the concepts from e-learning was disagreed by the majority of the respondents (42.6%, 40.6%, 60.2, and 38.3% respectively). Almost 40% felt that e-learning has moreover created problems rather than it has solved, 41.3% believed that it is

too time-consuming, 39.1% sensed that it had little impact on them and 31.5% said that it is not as informative as the teacher. Forty-three percent of the respondents agreed on e-learning replacing other forms of teaching and learning, 38.9% said that it helped them in reinforcing their knowledge, 45.4% felt that it helped them organize their work, and a half (50.4%) said they could easily catch up the missed lectures. Furthermore half (50.2%) of respondents agreed on e-learning increasing their effectiveness to create presentations and 58.9% on increasing their research capability. Similarly, 35.4% favored adopting e-learning by the universities for their students. The overall mean score for perceived usefulness was 3.06.

Table 3: Perceived usefulness

Statements		SD	DA	N	A	SA	Mean ± SD
**Perceived usefulness**							3.06 ± 0.54
1	E-learning can solve many of the educational problems.	31 (6.6)	77 (16.4)	176 (37.4)	136 (28.9)	50 (10.6)	3.21± 1.05
2	E-learning saves time.	21 (4.5)	38 (8.1)	107 (22.8)	192 (40.9)	112 (23.8)	3.71 ± 1.05
3	E-learning improves access to learning material.	36 (7.7)	71 (15.1)	172 (36.6)	143 (30.4)	48 (10.2)	3.20 ± 1.06
4	E-learning helps me to achieve better results.	68 (14.5)	132 (28.1)	188 (40.0)	67 (14.3)	15 (3.2)	2.64 ± 1.00
5	E-learning increase learner's engagement in learning.	47 (10.0)	144 (30.6)	165 (35.1)	95 (20.2)	19 (4.0)	2.78 ± 1.01
6	E- learning improve teacher and students interaction	116 (24.7)	167 (35.5.)	135 (28.7)	33 (7.0)	19 (4.0)	2.30 ± 1.04
7	E-learning increase my understanding of concept	55 (11.7)	125 (26.6)	199 (42.3)	71 (15.1)	20 (4.3)	2.74 ± 0.99
8	E-learning has created more problems than it solved	20 (4.3)	102 (21.7)	161 (34.3)	118 (25.1)	69 (14.7)	2.76 ± 1.08
9	E-learning is too time consuming to use.	52 (11.1)	142 (30.2)	158 (33.6)	75 (16.0)	43 (9.1)	3.18 ± 1.11
10	E-learning has had little impact on me	13 (2.8)	67 (14.3)	206 (43.8)	135 (28.7)	49 (10.4)	2.70 ± 0.93
11	E-learning is as informative as the teacher	30 (6.4)	118 (25.1)	177 (37.7)	111 (23.6)	34 (7.2)	3.00 ± 1.01
12	E-learning will never replace other forms of teaching and learning.	35 (7.4)	92 (19.6)	139 (29.6)	126 (26.8)	78 (16.6)	2.74 ± 1.16
13	E-learning help to reinforce my knowledge.	17 (3.6)	59 (12.6)	211 (44.9)	142 (30.2)	41 (8.7)	3.28 ± 0.91
14	E-learning help me to organize my work	9 (1.9)	65 (13.8)	183 (38.9)	161 (34.3)	52 (11.1)	3.39 ± 0.92
15	E-learning help me to catch up missed lectures	44 (9.4)	68 (14.5)	121 (25.7)	160 (34.0)	77 (16.4)	3.34 ± 1.18
16	E-learning increase my effectiveness to create presentations.	19(4.0)	73 (15.5)	142 (30.2)	137 (29.1)	99 (21.1)	3.48 ± 1.11

17	E-learning increase my research capability.	17 (3.6)	45 (9.6)	131 (27.9)	174 (37.0)	103 (21.9)	3.64 ± 1.03
18	Universities should adopt e-learning for their students.	53 (11.3)	73 (15.5)	178 (37.9)	115 (24.5)	51 (10.9)	3.08 ± 1.13

Intention to adopt e-learning

The majority (44.5%) of the respondents disagreed on e-learning making them uncomfortable and 33.2% on calling it a medium for dehumanizing the process of learning. Almost 45% of the respondents differed in the notion of disliking the idea of using e-learning and 31.1% were in favor of e-learning as it is not causing so much social isolation. About 40% of the respondents were interested in using e-learning, 35% already planned to participate in future e-learning courses, 24.7% were planning to buy a computer. Learning was fun through e-learning for 34.1%. The overall mean score for the intention to adopt e-learning was 3.07.

Table 4: Intention to adapt

Statements		SD	DA	N	A	SA	Mean ± SD
**Intention to adopt e-learning**							3.07 ± 0.69
1	E-learning makes me uncomfortable because I don’t understand it	55 (11.7)	154 (32.8)	145 (30.9)	78 (16.6)	38 (8.1)	3.23 ± 1.11
2	E-learning is a de-humanizing process of learning.	49 (10.4)	107(22.8)	194 (41.3)	85 (18.1)	35 (7.4)	3.11 ± 1.05
3	I dislike the idea of using E-learning.	80 (17.0)	131 (27.9)	149 (31.7)	74 (15.7)	36 (7.7)	3.31 ± 1.15
4	I am not in favor of E-learning as it leads to social isolation.	46 (9.8)	100 (21.3)	147 (31.3)	119 (25.3)	58 (12.3)	2.91 ± 1.16
5	E-learning doesn’t interest me.	51 (10.9)	136 (28.9)	157 (33.4)	87 (18.5)	39 (8.3)	3.16 ± 1.11
6	I plan to participate in future e-learning courses	43 (9.1)	88 (18.7)	172 (36.6)	119 (25.3)	48 (10.2)	3.09 ± 1.11
7	I plan to buy a computer to be able to follow lectures notes online	70 (14.9)	104 (22.1)	142 (30.2)	97 (20.6)	57 (12.1)	2.93 ± 1.22

8	Using E-learning makes learning fun.	46 (9.8)	81 (17.2)	183 (38.9)	124 (26.4)	36 (7.7)	3.05 ± 1.06
9	I don't know what I would do without E-learning.	53 (11.3)	115 (24.5)	168 (35.7)	95 (20.2)	39 (8.3)	2.91 ± 1.11

Ease of learning

For 45.5% of the respondents' use of e-learning was easier than using a library, 37.95% could easily use the web for lectures, 39.6% could learn the courses through the web, and acquiring any significant information by the internet was easy for 37.3% of the respondents. On the other hand, 47.2% felt that the use of the internet is making them slow and 54% said that technology can make them slaves sooner or later. The overall mean score for ease of e-learning use was 2.91.

Table 5: Ease of learning

Statements		SD	DA	N	A	SA	Mean ± SD
**Ease of learning**							2.91 ± 0.77
1	Using E-learning is more difficult than using the library	82 (17.4)	132 (28.1)	136 (28.9)	74 (15.7)	46 (9.8)	3.28 ± 1.21
2	I can't read the lectures notes through the web	51 (10.9)	127 (27)	136 (28.9)	86 (18.3)	70 (14.9)	3.01 ± 1.21
3	I can't learn courses through the web.	39 (8.3)	147 (31.3)	144 (30.6)	92 (19.6)	48 (10.2)	3.08 ± 1.11
4	It is difficult to acquire any significant information by using internet.	51 (10.9)	124 (26.4)	137 (29.1)	99 (21.1)	59 (12.6)	3.02 ± 1.18
5	It is difficult to express my thoughts by writing through E- learning.	23 (4.9)	74 (15.7)	148 (31.5)	157 (33.4)	68 (14.5)	2.63 ± 1.06
6	I find that using the internet make me slow	35 (7.4)	88 (18.7)	125 (26.6)	126 (26.8)	96 (20.4)	2.66 ± 1.21
7	I feel we are becoming slaves to technology.	31 (6.6)	48 (10.2)	137 (29.1)	136 (28.9)	118 (25.1)	2.44 ± 1.16
8	My interaction with E-learning is not understandable	38 (8.1)	134 (28.5)	186 (39.6)	77 (16.4)	35 (7.4)	3.13 ± 1.02

Technical support

The majority of the respondents had a neutral attitude towards technical support. Forty percent of the students had no idea regarding the updated website of their institute and only 28.1% seek any assistance from college support services. About thirty percent of students agreed on their institution providing e-learning training programs while 38.3% disagreed on their institution having adequate technology. The overall mean score for the provision of technical support for e-learning was 2.90.

Table 6: Technical support

1Statements		SD	DA	N	A	SA	Mean ± SD
**Technical support**							2.90 ± 0.81
1	My institute has an updated website.	58 (12.3)	85 (18.1)	188 (40.0)	107 (22.8)	32 (6.8)	2.94 ± 1.08
2	My institute facilitates e-learning training program	56 (11.9)	93 (19.8)	182 (38.7)	96 (20.4)	43 (9.1)	2.95 ± 1.11
3	My institute has adequate technology for e-learning.	66 (14.0)	114 (24.3)	166 (35.3)	91 (19.4)	33 (7.0)	2.81 ± 1.11
4	I seek technical assistance from college support services.	56 (11.9)	99 (21.1)	183 (38.9)	94 (20.0)	38 (8.1)	2.91 ± 1.09

Learning stressors

About thirty percent of the students felt anxious for their ability to use e-learning effectively, the majority 85.5% felt more stressed due to slow internet connection and 34.7% agreed on getting pressure from their teachers to use e-learning. The overall mean score for e-learning stressors was 2.53.

Table 7: Learning stressors

Statements	SD	DA	N	A	SA	Mean ± SD
**Learning stressor**						2.53 ± 0.69

1	Feel anxious about my ability to use e learning effectively.	39 (8.13)	111 (23.6)	177 (37.7)	106 (22.6)	37 (7.9)	3.02 ± 1.05
2	Slow internet connections stress me	9 (1.9)	18 (3.8)	41 (8.7)	95 (20.2)	307 (65.3)	1.57 ± 0.93
3	I feel pressured by my teachers to use E-learning for my research/ learning activities	67 (14.3)	107 (22.8)	133 (28.3)	95 (20.2)	68 (14.5)	3.02 ± 1.25

Distant use of e-learning

The majority (68.3%) supported the idea of e-learning as a medium to reach students living in remote areas and 66.7% agreed as it reduces the travel related stress. Regarding e-learning to be adapted to allow married students to balance family and study demands and to allow working students to study from home, it was agreed upon by 55.8% and 67.7% of the respondents respectively. The overall mean score for distant use of e-learning was 3.82.

Table 8: Distant use of e-learning

	**Statements**	**SD**	**DA**	**N**	**A**	**SA**	**Mean ± SD**
	**Distant use of e-learning**						3.82 ± 0.765
1	E-learning should be offered fully online to reach students living in remote areas.	24 (5.1)	29 (6.2)	96 (20.4)	134 (28.5)	187 (39.8)	3.92 ± 1.14
2	E-learning should be used to reduce travel related stress.	9 (1.9)	24 (5.1)	124 (26.4)	177 (37.7)	136 (28.9)	3.87 ± 0.95
3	E-learning should be adopted to allow married students to balance family and Study demands	26 (5.5)	37 (7.9)	145 (30.9)	156 (33.2)	106 (22.6)	3.59 ± 1.08
4	E-learning should be adopted to allow working students to study from home.	10 (2.1)	16 (3.4)	126 (26.8)	161 (34.3)	157 (33.4)	3.93 ± 0.96

The overall attitude of respondents regarding e-learning

Overall, 58.9% of the students had a positive attitude regarding e-learning. There was no statistical significance on the assessment of the association of attitude regarding e-learning with selected

sociodemographic variables like age, residence, college, year of study, and having been participated in e-learning earlier.

Table 6: Overall Attitude of the Respondents regarding e-learning (n=470)

Characteristics	Response			
	Favorable (≥ 60%)		Unfavorable (<60%)	
	Frequency	Percent (%)	Frequency	Percent (%)
**Overall Attitude**	277	58.9	193	41.1

Table 7: Association of attitude regarding e-learning with selected socio-demographic variables (n=470)

Characteristics	Categories	Attitude				p value*
		Negative Attitude		Positive Attitude		
		No.	%	No.	%	
**Age in years**	16-20<br>21-25<br>26-30	84<br>108<br>1	43.1<br>39.7<br>33.3	111<br>164<br>2	56.9<br>60.3<br>66.7	NA
**Residence**	Rural<br>Urban	34<br>159	43.0<br>40.7	45<br>232	57.0<br>59.3	0.71
**College**	PU<br>CMC, TU<br>CMS, KU<br>SMTC, PurU	68<br>45<br>34<br>46	46.9<br>35.7<br>34.0<br>46.5	77<br>81<br>66<br>53	53.1<br>64.3<br>66.0<br>53.5	0.07
**Year of study**	1st Year<br>2nd Year<br>3rd Year<br>4th Year	39<br>58<br>60<br>36	38.6<br>46.8<br>44.8<br>32.4	62<br>66<br>74<br>75	61.4<br>53.2<br>55.2<br>67.6	0.107
**Ever participated in e-learning before this pandemic**	Yes<br>No	14<br>179	50.0<br>77.1	28<br>249	66.7<br>58.2	0.28

Discussion

The sudden closure of all the educational institutions due to the pandemic caused the educational sector to seek alternative practices to limit the interference caused by the lockdown. The better way to deal with the situation came forward with the approach of practicing e-learning by every field of education to continue the teaching and learning process. The same occurred for the medical sector also where the students had to practice distant e-learning.

This study covers the nursing students' attitude regarding e-learning according to their experiences that they had during this pandemic throughout their learning activities. This study was conducted among the bachelor level nursing students, so it consisted only of female students with a mean age of 20.91 years which is similar to the study conducted in Indonesia among the medical students with a mean age of female students as 20.89 years. (11)

Mobile has been a popular gadget for e-learning as compared to laptops and computers. In this study, it was found that 76.45% of the respondents use mobile for their e-learning which is similar to the finding of a study conducted in Pakistan (76%). (12) However, in one of the studies conducted in India the use of mobile and laptop was approximately fifty-fifty. (13) Research conducted in Australia cited that the very common reason for the use of mobile was that learning can take place anytime and anywhere with the use of mobile. The majority (90.4%) of the respondents in the present study used Wi-Fi as a source of the internet rather than cellular data and telephone data which is similar to the study conducted in Nepal among the BDS students (81%). (14) The result of the same study was also similar to this study in the case of the percentage of respondents who had never participated in e-learning before this pandemic. In contrast to the finding of this study, one study had its majority of the students using data packs for their online class. (15)

Advantage and disadvantages of e-learning

The ability to stay at home was the major advantage of e-learning for the students in this study which was similar to the study conducted in Poland. (16) Apart from this, the reduction in the cost of accommodation and transport also remained the key benefit of e-learning in this study which is more similar to another study. (17)

Internet problems and technical issues were the major disadvantages of e-learning that the students experienced the most. These findings are parallel to the findings of other studies. (18) (15) (19) Furthermore lack of interaction with the patients was also stated as a disadvantage by almost 55% of the students. Learning from real patients in a clinical setting is crucial for nursing education and it cannot be fully replaced with distance learning. To some extent, a solution to this issue could be the use of virtual patients (VPs). VPs are designed to simulate real-life clinical scenarios and they enable the learner to prepare him/herself before a real patient encounter. (20) However, This finding is somewhat less than the finding of a study carried out among medical students of Pakistan. (20)

Effectiveness of e-learning to traditional face to face learning method

On the assessment of the effectiveness of e-learning over traditional learning**,** e-learning was found to be less effective than traditional face to face learning. The finding is consistent with the findings of many other studies conducted in India, Nepal, and Pakistan. (13)(21)(22) (5)The probable reason could be that the students are more acquainted with the traditional learning activities and since nursing involves the skill attainment tasks through practical, the objectives are not met through virtual learning. Another study conducted in Taiwan also indicated that face-to-face learning perception was higher than online learning in terms of all social presence, social interaction, and satisfaction.

Attitude of respondents regarding e-learning

About 59.6% of the students in this study had a positive perception of the usefulness of e-learning which is similar to the studies carried out by Ali N. et.al and Sharma K. (9) Regarding intention to adopt e-learning, almost all (100%) of the respondents wished-for adopting e-learning in near future whereas, in a study conducted in Pakistan, 85% wished to enroll and attend future e-learning courses. (9) In disparity to this finding, another study had only 23% of the respondents showing future e-learning preferences. (5) One study identified implementing distance e-learning in medical education is challenging as it could replace the theoretical knowledge but replacing the clinical medical skills is far behind. A mix of traditional and e-learning classes is the preferred way to deliver medical education in the future. (23)

Regarding ease of learning, only 51.3% had satisfactory responses while in contrast to this finding, the majority (79.2%) of the students in another study agreed as online applications are easy to use. Though almost half of the respondents believed that it's almost easy, the other 54% and 52% felt that they are becoming a slave to technology and that the internet is making them slow correspondingly.

In a study conducted in Iraq, lack of technical support was identified as one of the barriers to e-learning. It further pointed out that intensive training programs are required to enhance user skills towards computer and e-learning technologies. In addition to that, it suggested that there is an obvious lack of support from educational institutions to improve user confidence in adopting such technologies. As a consequence, users face difficulty with e-learning and this, in turn, may lead them to seek different reasons to avoid it. (18) In this study, technical support was the major neutral point for the students with a mean score of only 2.90. Findings of other studies also pointed out that insufficient technical support is a key challenge to fostering e-learning. (24,25)

Technical problems, such as an error in connection was the most important limitation to internet usage among medical students of Iran. (26) More than 85% of the respondents in this study were stressed with slow internet connections. Congruently, almost 30% felt anxious about their ability to use e-learning adding that to another level of stress.

Distance e-learning has been reported to provide easier and more effective access to a wider variety and greater quantity of information, as well, learning delivery allows a personalized approach in learning, so students have more control over the educational content, learning sequence, and time. (23) The present study showed a positive aspect of distance learning in the form of its ability to reach remote areas, reduction in the cost of travel, could be easily adapted by the married students to balance home and study (as there are females in majority of the nursing study) and as well to allow working students to study from home.

The mean scores for perceived usefulness, intention to adopt, and distant use of e-learning were 3.06, 3.07, and 3.82 which is much more in comparison to ease of learning, technical support, and learning stressors where the scores are 2.91, 2.90, and 2.53 respectively. The finding of our study is in line with the study conducted in Oman for the perceived usefulness (3.68), intention to adapt

(3.74), and distant use of e-learning (4.07) however lower for the other domains: learning stressors (3.25), technical support (3.05) and ease of learning (3.64).

In a study conducted in Iran to assess the attitude of medical students, 43.4% considered e-learning useful for medical education. (26) In this study, almost 59% of the students had a favorable attitude toward e-learning. The result was similar to the other studies conducted in Nepal. (27) (22) Another study conducted in Pakistan also showed a favorable attitude of the students. (5) Whereas, in a study conducted in India, the overall positive attitude was only from 30.8%.(13) A similar finding was found in another study also. (5) Furthermore, the overall satisfaction rate in medical distance learning was only 26.8% in one of the Jordanian universities, and it was significantly higher in students with previous experience in distance learning in their medical schools. (23)

There was no association of attitude of the respondents regarding e-learning with the selected socio-demographic variable (age, residence, college, year of study, and ever being participated in e-learning before this pandemic). The finding was not supported by the finding of a study which revealed the association of e-learning with residence, and family income. (28)

Limitations of the study

The nature of the study is the main limitation as the result cannot be generalized. Since the study has been conducted only with the nursing students of only 4 different nursing colleges, the findings cannot be generalized.

Conclusion

Although more than half of the respondents are positive regarding e-learning, students are more convinced for the usual traditional face to face learning than the virtual type. This can be due to practical issues. The nursing educational system should use the programs for improving e-learning that is more user friendly where virtual experiences of practical sessions can be carried out effectively and efficiently.

Availability of data and materials

The datasets used and analyzed during the current study are available from the corresponding author on reasonable request.

Abbreviations

CMC: Chitwan Medical Sciences

CMS: College of Medical Sciences

PU: Pokhara University

TU: Tribhuwan University

PurU: Purbanchal University

KU: Kathmandu University

SMTC: Shree Medical and Technical College

COVID-19: Corona Virus Disease 2019

SPSS: Statistical Package for Social Sciences

References

1. Dawadi S, Simkhada P. Impact of COVID-19 on the Education Sector in Nepal - Challenges and Coping Strategies. Sage Submissions [Internet]. 2020 [cited 2020 Jul 29];3(May):16. Available from: https://doi.org/10.31124/advance.12344336.v1

2. COVID-19: Higher Education challenges and responses - IAU [Internet]. [cited 2020 Jul 29]. Available from: https://iau-aiu.net/COVID-19-Higher-Education-challenges-and-responses

3. COVID-19 educational disruption and response: Continuation of radio education for secondary level students in Nepal [Internet]. [cited 2020 Jul 29]. Available from: https://en.unesco.org/news/covid-19-educational-disruption-and-response-continuation-radio-education-secondary-level

4. Effectiveness of online classes in Nepal: What three teachers felt after an experiment – OnlineKhabar [Internet]. [cited 2020 Jul 29]. Available from: https://english.onlinekhabar.com/effectiveness-of-online-classes-in-nepal-what-three-teachers-felt-after-an-experiment.html

5. Abbasi S, Ayoob T, Malik A, Memon SI. Perceptions of students regarding e-learning during covid-19 at a private medical college. Pakistan J Med Sci [Internet]. 2020 [cited

2020 Jul 29];36(COVID19-S4): S57–61. Available from: /pmc/articles/PMC7306963/?report=abstract

6. T M, S A, Aditya KS, Jha GK. Students' Perception and Preference for Online Education in India During COVID -19 Pandemic. SSRN Electron J [Internet]. 2020 May 13 [cited 2020 Jul 29]; Available from https://papers.ssrn.com/abstract=3596056

7. Dilemma And Difficulty Of Online Classes [Internet]. [cited 2020 Jul 29]. Available from: https://risingnepaldaily.com/opinion/dilemma-and-difficulty-of-online-classes

8. Innovating online education - my Republica - The New York Times Partner, Latest news of Nepal in English, Latest News Articles [Internet]. [cited 2020 Jul 29]. Available from: https://myrepublica.nagariknetwork.com/news/innovating-online-education/

9. Ali N, Jamil B, Sethi A, Ali S. ATTITUDE OF NURSING STUDENTS TOWARDS E-LEARNING. Vol. 2, Adv Health Prof Educ.

10. Vandana MEHRA A, Omidian F. DEVELOPMENT AN INSTRUMENT TO MEASURE UNIVERSITY STUDENTS' ATTITUDE TOWARDS E-LEARNING. Turkish Online Journal of Distance Education. 2012.

11. Daroedono E, Siagian FE, Alfarabi M, Cing JM, Arodes ES, Sirait RH, et al. The impact of COVID-19 on medical education: our students' perception on the practice of long-distance learning. Int J Community Med Public Heal [Internet]. 2020 Jun 26 [cited 2020 Jul 29];7(7):2790. Available from: http://www.ijcmph.com

12. Abbasi S, Ayoob T, Malik A, Memon SI. Perceptions of students regarding e-learning during covid-19 at a private medical college. Pakistan J Med Sci. 2020;36(COVID19-S4): S57–61.

13. Shete AN, Garkal KD, Somwanshi N, Professor A, Corresponding Author M. Perceptions of MBBS Students Regarding E-learning during COVID-19 Lockdown. Int J Heal Sci Res [Internet]. 2020 [cited 2020 Dec 18];10(9):319. Available from: www.ijhsr.org

14. Gupta A, Shrestha RM, Shrestha S, Acharya A, Pandey N. Perception of BDS students of

Kathmandu University on online learning during COVID-19 pandemic. Orthod J Nepal. 2020;10(2):20–8.

15. Subedi S, Nayaju S, Subedi S, Shah SK, Shah JM. Impact of E-learning during COVID-19 Pandemic among Nursing Students and Teachers of Nepal [Internet]. Vol. 5, International Journal of Science and Healthcare Research (www.ijshr.com). [cited 2020 Dec 20]. Available from: www.ijshr.com

16. MULYANI S. STUDENTS' PERCEPTION AND MOTIVATION TOWARD ENGLISH E-LEARNING DURING COVID-19 PANDEMIC (A STUDY AT THE TENTH GRADERS AT SMA N 1 SURUH IN THE ACADEMIC YEAR OF 2019/2020). 2020;

17. Esterhuyse M, Scholtz B, Venter D. Intention to use and satisfaction of e-learning for training in the corporate context. Interdiscip J Information, Knowledge, Manag. 2016;11:347–65.

18. Al-Azawei A, Parslow P, Lundqvist K. Barriers and Opportunities of E-Learning Implementation in Iraq: A Case of Public Universities. Vol. 17, International Review of Research in Open and Distributed Learning. 2016.

19. Almaiah MA, Al-Khasawneh A, Althunibat A. Exploring the critical challenges and factors influencing the E-learning system usage during COVID-19 pandemic. Educ Inf Technol [Internet]. 2020 Nov 1 [cited 2020 Dec 20];25(6):5261–80. Available from: /pmc/articles/PMC7243735/?report=abstract

20. Bączek M, Zagańczyk-Bączek M, Szpringer M, Jaroszyński A, Wożakowska-Kapłon B. Students' perception of online learning during the COVID-19 pandemic: a survey study of Polish medical students. 2020 Jul 15 [cited 2020 Jul 29]; Available from: https://doi.org/10.21203/rs.3.rs-41178/v1

21. Tuladhar SL, Pradhan D, Parajuli U, Manandhar P, Subedi N. Study on the effectiveness of online classes for undergraduate medical and dental students of Gandaki Medical College during COVID 19 pandemic period in Nepal. Orthod J Nepal [Internet]. 2020 Sep 11 [cited 2020 Dec 20];10(2):36–40. Available from:

https://www.nepjol.info/index.php/OJN/article/view/31146

22. Koirala D, Silwal M, Gurung S, Bhattarai M, Kumar V. Perception towards Online Classes during COVID-19 among Nursing Students of a Medical College of Kaski District, Nepal ABSTRACT *Corresponding author. 2020 [cited 2020 Dec 20]; Available from: https://dx.doi.org/10.37871/jbres1151

23. Al-Balas M, Al-Balas HI, Jaber HM, Obeidat K, Al-Balas H, Aborajooh EA, et al. Distance learning in clinical medical education amid COVID-19 pandemic in Jordan: Current situation, challenges, and perspectives. BMC Med Educ [Internet]. 2020 Oct 2 [cited 2020 Dec 21];20(1):341. Available from: https://bmcmededuc.biomedcentral.com/articles/10.1186/s12909-020-02257-4

24. Ssekakubo G, Suleman H, Marsden G. Issues of adoption: Have e-learning management systems fulfilled their potential in developing countries? In: ACM International Conference Proceeding Series [Internet]. New York, New York, USA: ACM Press; 2011 [cited 2020 Dec 21]. p. 231–8. Available from: http://dl.acm.org/citation.cfm?doid=2072221.2072248

25. Al-Shboul M. The level of E-learning integration at the University of Jordan: Challenges and opportunities. Int Educ Stud. 2013 Apr;6(4):93–113.

26. Ghanizadeh A. Use of E-learning in education: attitude of medical students of Shiraz, Iran. Int Med Med Investig J. 2018;3(3):108–11.

27. Sharma K, Deo G, Timalsina S, Joshi A, Shrestha N, Neupane HC. Online learning in the face of COVID-19 pandemic: Assessment of students' satisfaction at Chitwan medical college of Nepal. Kathmandu Univ Med J. 2020;18(19 70COVID-Special Issue):38–45.

28. Perception towards Online Classes during COVID-19 among Nursing Students of a Medical College of Kaski District, Nepal [Internet]. [cited 2020 Dec 21]. Available from: https://www.jelsciences.com/abstracts/918

Acknowledgments

We would like to thank all the participants who agreed to participate in this study and also all the associated medical colleges that provided us permission to collect the data.

Funding

This study did not receive any form of grants or financial support.

Author information

Affiliations

Maternal Health Nursing, College of Medical Sciences, Kathmandu University, Chitwan, Nepal

pratiimathapa@gmail.com

Pratima Thapa

Medical-surgical Nursing, Manmohan Memorial Institute of Health Sciences, Tribhuwan University, Kathmandu

Bhandarisuman1989@gmail.com

Suman Lata Bhandari

Medical-Surgical Nursing, Chitwan Medical College, Tribhuwan University, Chitwan, Nepal

Sharadapathak1@gmail.com

Sharada Pathak

Contributions

PT conceived the study, participated in the study design making process, carried out data collection, performed the statistical analysis, and drafted the manuscript. SLB and SP participated in data collection, statistical analysis, and finalized the manuscript. All authors read and approved the final manuscript.

Corresponding author

Pratima Thapa

Tel: +9771 9849884273

Email: pratiimathapa@gmail.com

Ethical Consideration

Formal permission was obtained from the Institutional Review Committee of the College of Medical Sciences- Teaching Hospital (COMSTH-IRC), Bharatpur-10 (Ref no. 2020-079, NHRC ref no. 2586).

Consent for publication

Not applicable

Competing interest

The authors declare that they have no competing interests.

Click here to access/download;Figure;Fig.docx

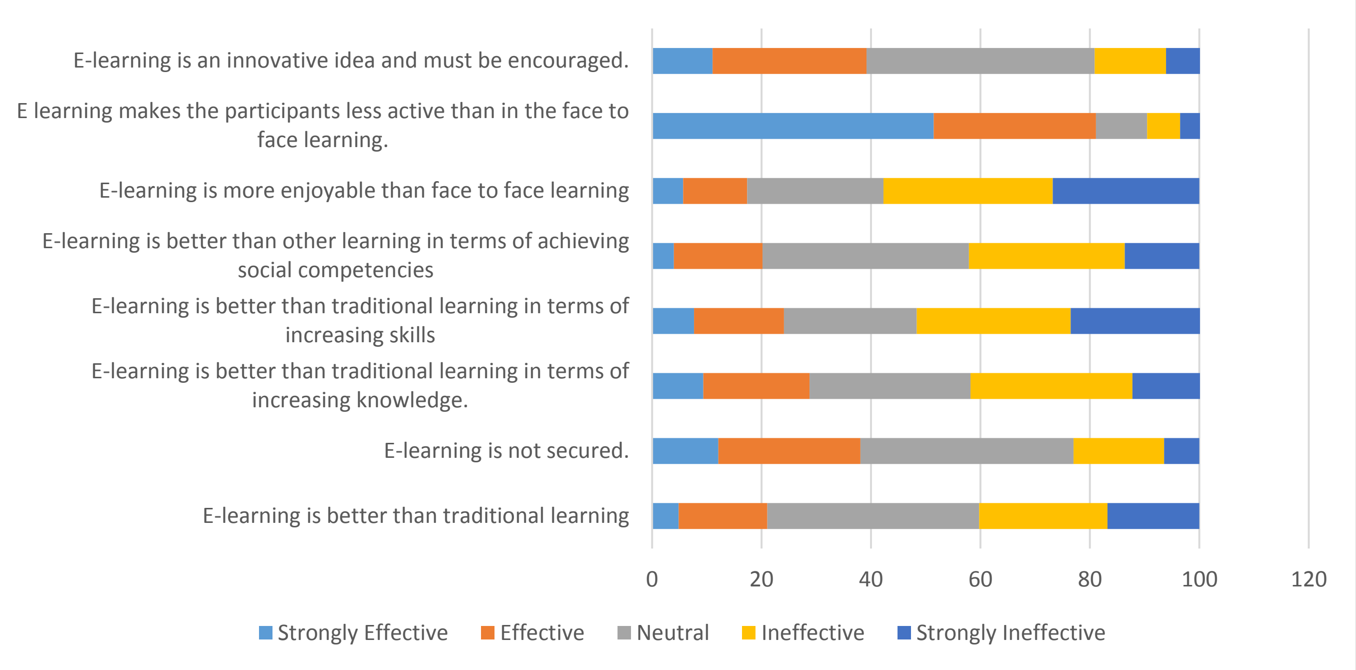

Fig- 1: Effectiveness of e-learning in relation to traditional face to face learning method (n=470)
