# Supplementary material for: Nursing students’ attitude on the practice of e-learning: A cross-sectional survey amid COVID-19 in Nepal
Source: PLoS One. 2021 Jun 24;16(6):e0253651. doi: 10.1371/journal.pone.0253651 (PMC8224981; doi:10.1371/journal.pone.0253651)
Supplement: Attachment — Submitted filename: Review.docx [file pone.0253651.s003.docx]

Page 2 – suggest using cessation or another word instead of shutting down, please edit throughout.

Page 3 please remove etc from the paper entirely and list the extra problems

Page 3 there appears to be different fonts used at times?

Page 3 suggest editing this sentence please So, based on this the research team ~~researcher~~ was ~~is~~ interested in carrying out a study regarding ~~the attitude of~~ ~~the~~ nursing students’ attitudes towards e-learning

Page 3 I understand English may not be the author’s first language and you are to be congratulated for writing this paper fully in English, that must be difficult at times. However the readership will expect correct English grammar and punctuation. Could you have someone go over the paper please and correct statements such as this was never been tried before resubmitting. I will not comment any further on grammar and punctuation however I have highlighted some in the attached PDF.

Methods – please reference and provide more information on the study method chosen, the validity testing and reporting of the statistical analysis of the survey in some more depth. Using the equator website is recommended and the tools there will assist you to include relevant information in the paper. For cross-sectional studies you could use the STROBE tool.

Please remove all abbreviations such as B.Sc.

Page 4 the phases of the study could be moved into a table.

I don’t think Ethical consideration: should be included please write this in a full sentence.

The results section has a lot of tables and data reported is not discussed in the discussion section. I would reconsider the important or new or interesting results and present and report only on them.

The discussion also has some paragraphs that would be better placed in the background section for example the two that begin In a study conducted in Iraq……

This paragraph is repetitive of the results section and should not be in the discussion The mean scores for perceived usefulness, intention to adopt, and distant use of e-learning were

3.06, 3.07, and 3.82 which is much more in comparison to ease and there are other paragraphs similar to this one.

The conclusion in the paper is very short and seems to generalise the paper in the international data. For example there are many nursing education providers that do and have shown effective, reliable and efficient use of e-learning and virtual learning experiences. I think overall a wider literature review and a more honed problem statements is needed for this paper to progress to publication.

Also regarding the referencing the numbers need to be super script like so ^1^

There does appear to be some inconsistencies in the reference list such as some doi included and different presentations of the doi. Also see number 24 I am not aware of using cited as in the reference list, however I am not an expert on Vancouver style so I stand corrected if this is normal practice.
