# Supplementary material for: Nursing students’ attitude on the practice of e-learning: A cross-sectional survey amid COVID-19 in Nepal
Source: PLoS One. 2021 Jun 24;16(6):e0253651. doi: 10.1371/journal.pone.0253651 (PMC8224981; doi:10.1371/journal.pone.0253651)
Supplement: Attachment — Submitted filename: PONE-D-21-00551-R2R.docx [file pone.0253651.s005.docx]

**Author’s response to academic editor and reviewers**

Title: Nursing students’ attitude on the practice of e-learning: A cross-sectional survey amid Covid 19 in Nepal

Authors:

Pratima Thapa ([pratiimathapa@gmail.com](mailto:pratiimathapa@gmail.com))

Suman Lata Bhandari (bhandarisuman1989@gmail.com)

Sharada Pathak ([sharadapathak1@gmail.com](mailto:sharadapathak1@gmail.com))

Comments for authors

1. Please ensure that your manuscript meets PLOS ONE's style requirements, including those for file naming. The PLOS ONE style templates can be found at

<https://journals.plos.org/plosone/s/file?id=wjVg/PLOSOne_formatting_sample_main_body.pdf> and

<https://journals.plos.org/plosone/s/file?id=ba62/PLOSOne_formatting_sample_title_authors_affiliations.pdf>

*We are very grateful for the academic editor’s suggestions. The manuscript has been modified as per the PLOS One’s style requirement, including for the file naming. The template has been followed.*

2. Thank you for submitting the above manuscript to PLOS ONE. During our internal evaluation of the manuscript, we found significant text overlap between your submission and the following previously published works, some of which you are an author.

Introduction section:
- <https://www.researchgate.net/publication/342501833_The_impact_of_COVID-19_on_Education_in_Ghana>
- <https://www.jelsciences.com/articles/jbres1151.php>
- <http://www.pjms.org.pk/index.php/pjms/article/view/2766>

Results & Discussion sections:
- <https://www.semanticscholar.org/paper/Attitude-of-nursing-students-towards-e-learning-Ali-Jamil/6349db2f9f5bb9e1fcb06b7a84b423b47c713c13?p2df>
- <https://www.researchsquare.com/article/rs-41178/v1>
- <https://www.erudit.org/fr/revues/irrodl/2016-v17-n5-irrodl04876/1064708ar.pdf>
- <https://bmcmededuc.biomedcentral.com/articles/10.1186/s12909-020-02257-4>

We would like to make you aware that copying extracts from previous publications, especially outside the methods section, word-for-word is unacceptable. In addition, the reproduction of text from published reports has implications for the copyright that may apply to the publications.

Please revise the manuscript to rephrase the duplicated text, cite your sources, and provide details as to how the current manuscript advances on previous work. Please note that further consideration is dependent on the submission of a manuscript that addresses these concerns about the overlap in text with published work.

We will carefully review your manuscript upon resubmission, so please ensure that your revision is thorough.

*Manuscript has been revised and cited for the sources. Rationale for the conduction of the research has also been revised.*

3. Please include additional information regarding the survey or questionnaire used in the study and ensure that you have provided sufficient details that others could replicate the analyses.

For instance, if you developed the survey or questionnaire as part of this study and it is not under a copyright more restrictive than CC-BY, please include a copy, in both the original language and English, as Supporting Information.

If the questionnaire is published, please provide a citation to the (i) questionnaire and/or (ii) original publication associated with the questionnaire.

*Additional information regarding the survey and questionnaire has been provided.*

*The question has four parts. Part 1, 2 and 3 was developed by the authors themselves, and part 4 was obtained from previous study. Necessary permission was obtained from the author of the previous study to use the questionnaire in part 4 from the authors. Citation has been provided in the manuscript.*

*The questionnaire has been provided as a separate file as supporting information.*

4. We note that you have indicated that data from this study are available upon request. PLOS only allows data to be available upon request if there are legal or ethical restrictions on sharing data publicly. For information on unacceptable data access restrictions, please see <http://journals.plos.org/plosone/s/data-availability#loc-unacceptable-data-access-restrictions>.

In your revised cover letter, please address the following prompts:

a) If there are ethical or legal restrictions on sharing a de-identified data set, please explain them in detail (e.g., data contain potentially identifying or sensitive patient information) and who has imposed them (e.g., an ethics committee). Please also provide contact information for a data access committee, ethics committee, or other institutional body to which data requests may be sent.

b) If there are no restrictions, please upload the minimal anonymized data set necessary to replicate your study findings as either Supporting Information files or to a stable, public repository and provide us with the relevant URLs, DOIs, or accession numbers. Please see <http://www.bmj.com/content/340/bmj.c181.long> for guidelines on how to de-identify and prepare clinical data for publication. For a list of acceptable repositories, please see <http://journals.plos.org/plosone/s/data-availability#loc-recommended-repositories>.

We will update your Data Availability statement on your behalf to reflect the information you provide.

*Data file has been made available in the supporting information files.*

5. We note you have included ten tables to which you do not refer in the text of your manuscript. We also note that there are 8 table numbers in titles and table no.s 6 and 7 are duplicated in table titles.

Please ensure that you refer to all Tables in your text; if accepted, production will need this reference to link the reader to each Table.

*The table number has been corrected and all tables has been referred in the text.*

5. Your ethics statement should only appear in the Methods section of your manuscript. If your ethics statement is written in any section besides the Methods, please delete it from any other section.

*Ethics statement from other section has been removed.*

Additional Editor Comments

Reviewers' comments:

Reviewer's Responses to Questions

**Comments to the Author**

1. Is the manuscript technically sound, and do the data support the conclusions?

The manuscript must describe a technically sound piece of scientific research with data that supports the conclusions. Experiments must have been conducted rigorously, with appropriate controls, replication, and sample sizes. The conclusions must be drawn appropriately based on the data presented.

Reviewer #1: Yes

Reviewer #2: Partly

*Manuscript has been modified as per the given suggestions and feedbacks.*

2. Has the statistical analysis been performed appropriately and rigorously?

Reviewer #1: I Don't Know

Reviewer #2: No

*Statistical portion has been changed.*

3. Have the authors made all data underlying the findings in their manuscript fully available?

The [PLOS Data policy](http://www.plosone.org/static/policies.action#sharing) requires authors to make all data underlying the findings described in their manuscript fully available without restriction, with rare exception (please refer to the Data Availability Statement in the manuscript PDF file). The data should be provided as part of the manuscript or its supporting information, or deposited to a public repository. For example, in addition to summary statistics, the data points behind means, medians and variance measures should be available. If there are restrictions on publicly sharing data—e.g. participant privacy or use of data from a third party—those must be specified.

Reviewer #1: No

Reviewer #2: Yes

*Data has been provided as supporting information files.*

4. Is the manuscript presented in an intelligible fashion and written in standard English?

PLOS ONE does not copyedit accepted manuscripts, so the language in submitted articles must be clear, correct, and unambiguous. Any typographical or grammatical errors should be corrected at revision, so please note any specific errors here.

Reviewer #1: No

Reviewer #2: Yes

*Language has been corrected.*

5. Review Comments to the Author

Please use the space provided to explain your answers to the questions above. You may also include additional comments for the author, including concerns about dual publication, research ethics, or publication ethics. (Please upload your review as an attachment if it exceeds 20,000 characters)

Reviewer #1: Dear Authors
thank you for the time you have taken for undertaking both this study and the committment to writing the manuscript. I think the paper could add to the body of literature around e-learning challenges during COVID however this study needs to be situated in the Nepalese context and the international literature. The methods section requires more work to meet the PLOS guidelines of "which focus on the technical aspects of a study rather than more subjective evaluations of issues like 'impact' or 'interest level' and I have made comments in the word document about this. Please review the attached documents that may assist you to strengthen the paper. I wish you well in publishing your work.

*Thank you for your constructive feedback*. *The revision has been done as per the comments below.*

Page 2 – suggest using cessation or another word instead of shutting down, please edit throughout.

*Shutting down word has been edited with other words throughout the manuscript*

Page 3 please remove etc from the paper entirely and list the extra problems

*Etc has been removed*

Page 3 there appears to be different fonts used at times?

*Fonts has been made uniform. Font styles are used as per the guidelines of plos one.*

Page 3 suggest editing this sentence please So, based on this the research team ~~researcher~~ was ~~is~~ interested in carrying out a study regarding ~~the attitude of~~ ~~the~~ nursing students’ attitudes towards e-learning

*Edit has been done as per the suggestion.*

Page 3 I understand English may not be the author’s first language and you are to be congratulated for writing this paper fully in English, that must be difficult at times. However the readership will expect correct English grammar and punctuation. Could you have someone go over the paper please and correct statements such as this was never been tried before resubmitting. I will not comment any further on grammar and punctuation however I have highlighted some in the attached PDF.

*Thank you for the feedback. I have tried my best to correct English grammar in the revised manuscript.*

Methods – please reference and provide more information on the study method chosen, the validity testing and reporting of the statistical analysis of the survey in some more depth. Using the equator website is recommended and the tools there will assist you to include relevant information in the paper. For cross-sectional studies you could use the STROBE tool.

*It has been revised.*

Please remove all abbreviations such as B.Sc.

*Removed all abbreviations.*

Page 4 the phases of the study could be moved into a table.

I don’t think Ethical consideration: should be included please write this in a full sentence.

*Ethical consideration has been written in full sentence.*

The results section has a lot of tables and data reported is not discussed in the discussion section. I would reconsider the important or new or interesting results and present and report only on them.

*Important and new findings are discussed in the discussion.*

The discussion also has some paragraphs that would be better placed in the background section for example the two that begin In a study conducted in Iraq……

*Background section has been provided with the data.*

This paragraph is repetitive of the results section and should not be in the discussion The mean scores for perceived usefulness, intention to adopt, and distant use of e-learning were

3.06, 3.07, and 3.82 which is much more in comparison to ease and there are other paragraphs similar to this one.

*This has been removed.*

The conclusion in the paper is very short and seems to generalise the paper in the international data. For example there are many nursing education providers that do and have shown effective, reliable and efficient use of e-learning and virtual learning experiences. I think overall a wider literature review and a more honed problem statements is needed for this paper to progress to publication.

*Conclusion has been re-written.*

Also regarding the referencing the numbers need to be super script like so ^1^

The references are made as per the feedback.

There does appear to be some inconsistencies in the reference list such as some doi included and different presentations of the doi. Also see number 24 I am not aware of using cited as in the reference list, however I am not an expert on Vancouver style so I stand corrected if this is normal practice.

*Vancouver style referencing list has been corrected.*

Reviewer #2: Although the manuscript describes a technically sound piece of scientific research with data which support the conclusions, the research methodology is too simplistic and the weak descriptive findings reported in the manuscript contribute little to existing knowledge. Many similar research studies (e.g. Regmi & Regmi, 2010) have been conducted.
Regmi, K. R., & Regmi, S. (2010). Medical and nursing students attitudes towards interprofessional education in Nepal. Journal of Interprofessional Care, 24(2), 150–167. <https://doi.org/10.3109/13561820903362254>.

A similarity test reported an index of 43%. The author is suggested to ensure this manuscript is free from any issues of plagiarism.

In addition to providing descriptive statistics and identifying statistical relationships between the five attitudinal dimensions and demographic variables, the author can consider exploring the statistical relationships among the five dimensions and overall attitudes. Statistical tests such as correlations, multiple regression, ANOVA and MANOVER can be considered.

Overall, the manuscript suffers from a lack of contribution of the research study reported and the simplistic research method.

*Correlation has been established between the domains and also with the overall attitude regarding e-learning.*

6. PLOS authors have the option to publish the peer review history of their article ([what does this mean?](https://journals.plos.org/plosone/s/editorial-and-peer-review-process#loc-peer-review-history)). If published, this will include your full peer review and any attached files.


If you choose “no”, your identity will remain anonymous but your review may still be made public.


**Do you want your identity to be public for this peer review?** For information about this choice, including consent withdrawal, please see our [Privacy Policy](https://www.plos.org/privacy-policy).

Reviewer #1: No

Reviewer #2: No

[NOTE: If reviewer comments were submitted as an attachment file, they will be attached to this email and accessible via the submission site. Please log into your account, locate the manuscript record, and check for the action link "View Attachments". If this link does not appear, there are no attachment files.]

While revising your submission, please upload your figure files to the Preflight Analysis and Conversion Engine (PACE) digital diagnostic tool, <https://pacev2.apexcovantage.com/>. PACE helps ensure that figures meet PLOS requirements. To use PACE, you must first register as a user. Registration is free. Then, login and navigate to the UPLOAD tab, where you will find detailed instructions on how to use the tool. If you encounter any issues or have any questions when using PACE, please email PLOS at [figures@plos.org](mailto:figures@plos.org). Please note that Supporting Information files do not need this step.

*Yes, the figures have been done as per the guidelines.*
